# Hepatic Artery Injection of ^131^I-Metuximab Combined with Transcatheter Arterial Chemoembolization for Unresectable Hepatocellular Carcinoma: A Prospective Nonrandomized, Multicenter Clinical Trial

**DOI:** 10.2967/jnumed.121.262136

**Published:** 2022-04

**Authors:** Hui Chen, Gang Nan, Ding Wei, Ren-You Zhai, Ming Huang, Wu-Wei Yang, Bao-Cai Xing, Xu Zhu, Hai-Feng Xu, Xiao-Dong Wang, Xiao-Yong Zhang, Bao-Rang Zhu, Peng Liu, Guang Cao, Song Gao, Chun-Yi Hao, Ren-Jie Yang, Jian-Hai Guo, Xin Zhang, Kun Gao, Kun Wang, Jian-Feng Wang, Zi-Yu Li, Lin-Zhong Zhu, Rong Ding, Jing Li, Ling Zhao, Yu-Jun Shao, Hai-Chun Liu, Jie-Lai Xia, Ling Wang, Ling-Min Kong, Zhi-Nan Chen, Huijie Bian

**Affiliations:** 1 Key Laboratory of Carcinogenesis and Translational Research, Peking University Cancer Hospital, Beijing, China;; 2 National Translational Science Center for Molecular Medicine and Department of Cell Biology, State Key Laboratory of Cancer Biology, Fourth Military Medical University, Xi’an, China;; 3 Beijing Chao-Yang Hospital, Capital Medical University, Beijing, China;; 4 Yunnan Cancer Hospital, Third Affiliated Hospital of Kunming Medical University, Kunming, China;; 5 Fifth Medical Center, Chinese PLA General Hospital, Beijing, China;; 6 China Nuclear Industry Beijing 401 Hospital, Beijing, China; and; 7 College of Military Preventive Medicine, Fourth Military Medical University, Xi’an, China

**Keywords:** ^131^I-labeled metuximab, transcatheter arterial chemoembolization, hepatocellular carcinoma

## Abstract

This prospective nonrandomized, multicenter clinical trial was performed to investigate the efficacy and safety of ^131^I-labeled metuximab in adjuvant treatment of unresectable hepatocellular carcinoma. **Methods:** Patients were assigned to treatment with transcatheter arterial chemoembolization (TACE) combined with ^131^I-metuximab or TACE alone. The primary outcome was overall tumor recurrence. The secondary outcomes were safety and overall survival. **Results:** The median time to tumor recurrence was 6 mo in the TACE + ^131^I-metuximab group (*n* = 160) and 3 mo in the TACE group (*n* = 160) (hazard ratio, 0.55; 95% CI, 0.43–0.70; *P* < 0.001). The median overall survival was 28 mo in the TACE + ^131^I-metuximab group and 19 mo in the TACE group (hazard ratio, 0.62; 95% CI, 0.47–0.82; *P* = 0.001). **Conclusion:** TACE + ^131^I-metuximab showed a greater antirecurrence benefit, significantly improved the 5-y survival of patients with advanced hepatocellular carcinoma, and was well tolerated by patients.

## Visual Abstract

**Figure:**
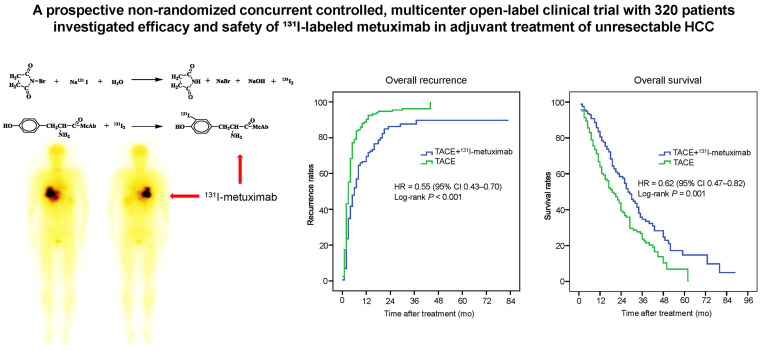

Hepatocellular carcinoma (HCC) is the sixth most common malignancy and the third leading cause of cancer-related death worldwide (*1*). Systematic treatment for advanced HCC remains of great concern (*2*). Although transcatheter arterial chemoembolization (TACE) is frequently used for the treatment of HCC, it fails to lead to a complete response in most patients, especially in the middle or late stage when the tumor is larger than 5 cm. ^131^I-metuximab is a radioimmunoconjugate generated by labeling metuximab directed against CD147, which is associated with hepatocarcinogenesis and tumor metastasis (*3*,*4*). Previous studies have shown the beneficial treatment effects of ^131^I-metuximab combined with TACE in patients with HCC, and no severe toxicities were reported in these studies (*5*,*6*). In this study, we conducted a prospective clinical trial to evaluate the therapeutic efficacy of ^131^I-metuximab combined with TACE in patients with unresectable HCC.

## MATERIALS AND METHODS

### Patient Population and Study Design

Between November 2, 2011, and December 31, 2015, a prospective, nonrandomized concurrent controlled, multicenter, open-label clinical trial was performed on patients with unresectable HCC at 4 medical centers in China. Patients diagnosed with unresectable HCC according to the guidelines of the American Association for the Study of Liver Diseases were assigned to the TACE + ^131^I-metuximab or TACE group (*7*). To minimize bias, we matched patients between the 2 groups based on age, sex, Barcelona Clinic Liver Cancer stage, Child–Pugh class, and Eastern Cooperative Oncology Group score. The Medicine Ethics Committee of Peking University Cancer Hospital approved this study, and all subjects gave written informed consent. The study was registered at http://www.chictr.org.cn/ (ChiCTR-ONRC-11001664).

### Inclusion and Exclusion Criteria

The eligibility criteria included men and women aged 18–80 y, with confirmed HCC according to the criteria of the American Association for the Study of Liver Diseases, Barcelona Clinic Liver Cancer stage B or C, Eastern Cooperative Oncology Group performance status of no more than 2, Child–Pugh liver function class A or B, platelet count of at least 70 × 10^9^ per liter, white blood cell count of at least 3 × 10^9^ per liter, no organ dysfunction, and a life expectancy of at least 3 mo. Patients who were allergic to biologic products, pregnant, or lactating or had thyroid hypofunction, brain metastases, or a positive initial skin test for metuximab were excluded. The selection criteria and algorithm used to determine patient grouping were completely and strictly consistent across different centers.

### Drugs and Treatments

The patients in both groups underwent standard TACE treatment according to the Clinical Guidelines for the Diagnosis and Treatment of Primary Liver Cancer, China, 2011. For TACE administration, a catheter was placed into the proper hepatic artery through the femoral artery using the Seldinger technique. For hepatic lesions with a rich blood supply, hepatic arterial chemoembolization was conducted first (pharmorubicin [40 mg] and ultra-fluid lipiodol [3–20 mL] were administered according to the tumor size). After embolization, 750 mg of diluted 5-fluorouracil was perfused via a 2.4-F microcatheter. In the TACE + ^131^I-metuximab group, patients were transferred to the nuclear medicine ward after TACE, and a 27.75 MBq/kg dose of ^131^I-metuximab was administered into the proper hepatic artery (*8*).

### Sample Size

According to our previous research, the assumptions were a 1-y recurrence rate of 50% in the TACE + ^131^I-metuximab group and 69.5% in the TACE group. We needed 141 patients in each group (power of 90%, 2-sided significance level of 5%, 1:1 allocation) to detect a 19.5% difference in recurrence rate between groups. We also estimated and added 10% to account for patients who might have been lost to follow-up. On the basis of these calculations, we estimated that we needed to enroll at least 155 patients.

### Outcomes and Evaluation

The primary outcome was overall tumor recurrence, which was measured from the date of the first TACE after allocation until the first documented tumor recurrence event and based on the assessment criteria of the modified Response Evaluation Criteria in Solid Tumors. The secondary outcomes were safety and overall survival. Safety was assessed according to the National Cancer Institute’s Common Terminology Criteria for adverse effects (version 4.0).

### Statistical Analysis

Overall recurrence and overall survival were analyzed using the Kaplan–Meier method and a log-rank test with a 2-sided overall α-level of 0.05. *P* values were 2-sided, and less than 0.05 was considered statistically significant. Statistical analyses were performed using SPSS software (version 16.0; IBM Corp.).

## RESULTS

**FIGURE 1.**
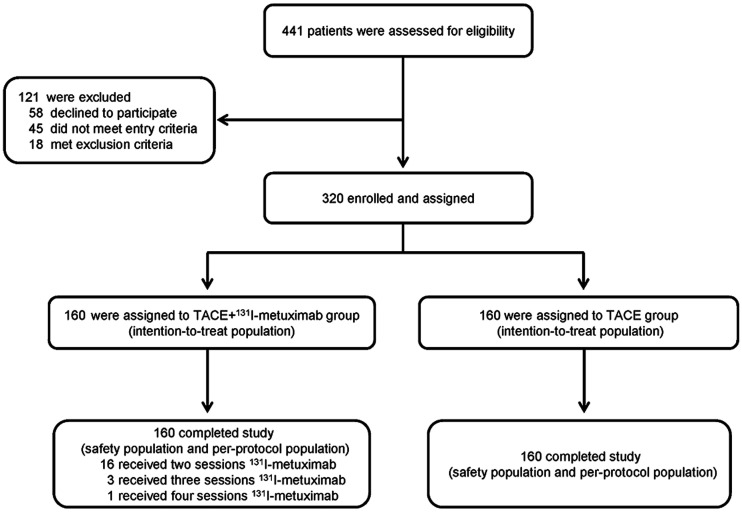
Trial profile: enrollment and outcomes.

We evaluated 441 Chinese patients with a confirmed diagnosis of HCC. On the basis of the inclusion and exclusion criteria, 320 patients were enrolled in our study, with 160 (50%) patients assigned to the TACE + ^131^I-metuximab group and 160 (50%) patients assigned to the TACE group (Fig. 1). Baseline patient characteristics are shown in Supplemental Tables 1 and 2 (supplemental materials are available at http://jnm.snmjournals.org).

**FIGURE 2.**
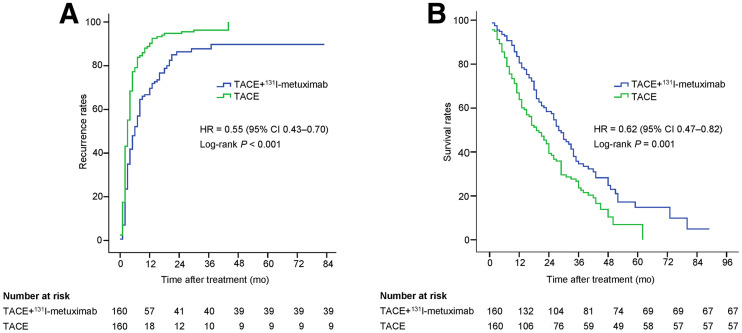
Overall recurrence (A) and overall survival (B) data analyzed using Kaplan–Meier method and log-rank test. HR = hazard ratio.

**FIGURE 3.**
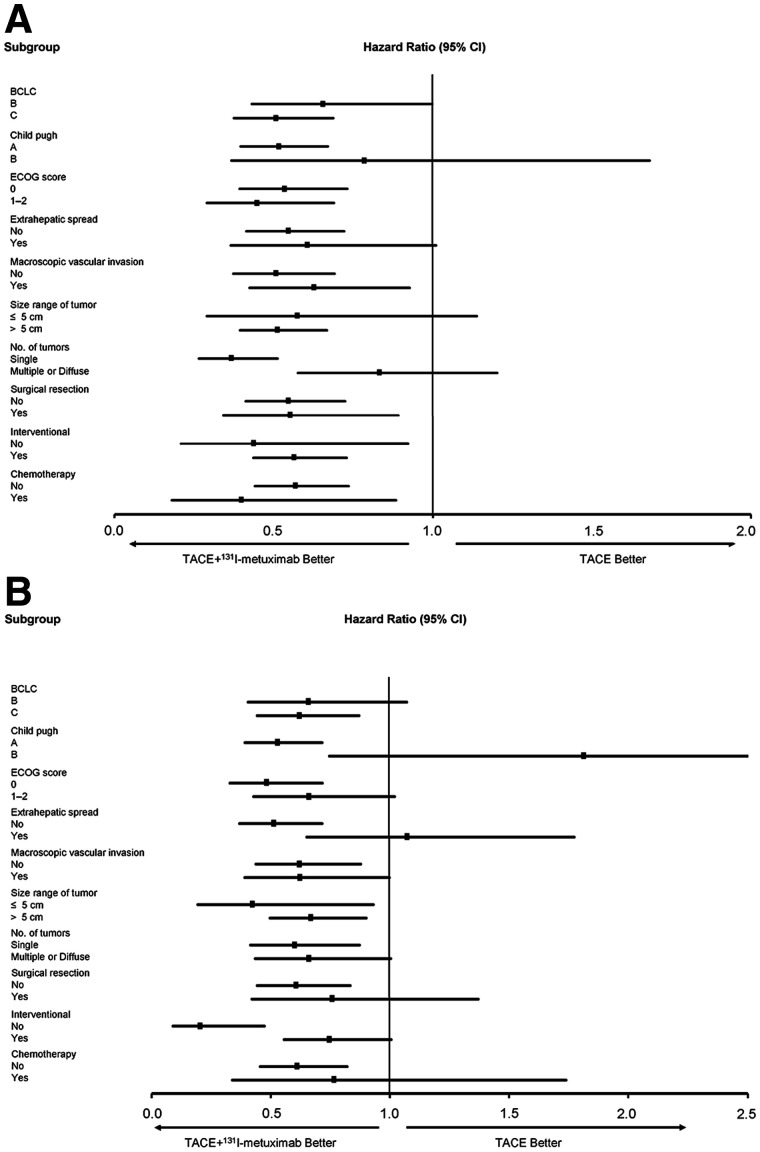
Analysis of overall recurrence (A) and overall survival (B) in selected subgroups according to baseline prognostic factors, performed using Cox regression models. BCLC =Barcelona Clinic Liver Cancer; ECOG = Eastern Cooperative Oncology Group.

The study was completed on March 30, 2020. The median follow-up period was 17 mo (interquartile range, 8–30 mo). At that time, 121 (76%) patients in the TACE + ^131^I-metuximab group and 151 (94%) patients in the TACE group had developed tumor recurrence. In the TACE + ^131^I-metuximab group, 100 (63%) patients had new intrahepatic recurrence, 102 (64%) patients had intrahepatic residual recurrence, and 52 (33%) patients had extrahepatic metastasis, compared with 128 (80%), 130 (81%), and 98 (61%) patients, respectively, in the TACE group—a significant difference between the 2 groups (Supplemental Table 3). The median time to overall tumor recurrence was significantly longer in the TACE + ^131^I-metuximab group than in the TACE group (6 vs. 3 mo; hazard ratio, 0.55; 95% CI, 0.43–0.70; *P* < 0.001). The log-rank test revealed a significant difference in the recurrence rates between the 2 groups (*P* < 0.001) (Fig. 2A). The significant antirecurrence benefits represented a relative reduction of 23% in tumor recurrence at 12 mo (Supplemental Table 4). An exploratory multivariate analysis using the Cox proportional-hazards model identified 7 baseline characteristics that were prognostic indicators of overall tumor recurrence. After adjusting for these prognostic factors, the effect of ^131^I-metuximab on overall recurrence remained significant (hazard ratio, 0.46; 95% CI, 0.35–0.61; *P* < 0.001). A prespecified subgroup analysis showed an antirecurrence benefit for TACE + ^131^I-metuximab over TACE alone in most of the subgroups analyzed (Fig. 3A).

At the time of the final analysis, 93 (58%) patients in the TACE + ^131^I-metuximab group and 113 (71%) patients in the TACE group had died. The median overall survival was significantly longer in the TACE + ^131^I-metuximab group than in the TACE group (28 vs. 19 mo; hazard ratio, 0.62; 95% CI, 0.47–0.82; *P* = 0.001). The log-rank test revealed a significant difference in survival rate between the 2 groups (*P* = 0.001) (Fig. 2B). The survival rates are shown in Supplemental Table 4. An exploratory multivariate analysis using the Cox proportional-hazards model identified 7 baseline characteristics that were prognostic indicators of overall survival. After adjusting for these prognostic factors, the effect of TACE + ^131^I-metuximab on overall survival was significantly different from that of TACE alone (hazard ratio, 0.55; 95% CI, 0.41–0.74; *P* < 0.001). A prespecified subgroup analysis showed a survival benefit for TACE + ^131^I-metuximab over TACE alone in Barcelona Clinic Liver Cancer stage C, Child–Pugh class A, Eastern Cooperative Oncology Group score 0, extrahepatic spread (no), macroscopic vascular invasion (no), size range of tumor, number of tumors (single), and previous therapy (no) subgroups (Fig. 3B).

The reported adverse effects for patients receiving TACE + ^131^I-metuximab were predominantly grade 1 or 2 in constitutional symptoms and gastrointestinal events such as fever, pain, vomiting, and fatigue (Supplemental Table 5). Grade 3 laboratory abnormalities included a decrease in white blood cell count (5% in the TACE + ^131^I-metuximab group vs. 0.6% in the TACE group, *P* = 0.04). No serious adverse effects and treatment-related deaths were observed. Taken together, the descriptive data suggested that ^131^I-metuximab as a radioimmunotherapeutic agent did not pose a hazard to hepatic function in the TACE + ^131^I-metuximab group.

At the time of analysis, 206 (64%) patients in the 2 groups had died. A total of 93 (58%) patients in the TACE + ^131^I-metuximab group died, and 113 (71%) patients in the TACE group died. The causes of death are shown in Supplemental Table 6. The χ^2^ test showed a significant difference between the 2 groups (*P* = 0.018).

## DISCUSSION

TACE is still an important therapy for unresectable HCC, but the median recurrence time is reported to be 3 mo (*9*). Our study indicated that ^131^I-metuximab combined with TACE delayed tumor recurrence by 3 mo in patients with unresectable HCC and preserved liver function compared with TACE alone. The results also demonstrated that the TACE group had a higher risk of recurrence and extrahepatic metastasis, and, especially, early recurrence, relative to the TACE + ^131^I-metuximab group, suggesting that TACE alone could manage the existing intrahepatic tumor and that ^131^I-metuximab could inhibit tumor recurrence and metastasis.

In this study, tumor parenchyma was embolized with lipiodol instead of particles, whereas the main tumor-supplying artery was preserved. Therefore, the formation of tortuous blood circulation in the tumor was relatively less, as was conducive with subsequent interventional therapy. For ethical reasons, we did not use ^131^I-metuximab alone for comparison. Because of the radioactivity of the drug, we could not perform a double-blind study for the safety of patients and doctors. This study had several limitations, including mixed populations of previously treated and untreated individuals and a lack of double blinding and randomization, which may result in a subjective bias. In addition, when recurrence was detected, the fact that patients were treated with various treatments may affect the overall survival results. Nevertheless, caution should be exercised when analyzing the results of a nonrandomized concurrent control trial; well-designed prospective, randomized, controlled trials remain necessary.

## CONCLUSION

The combination of TACE and ^131^I-metuximab represents a logical, new, and encouraging approach to neoadjuvant therapy for advanced HCC. ^131^I-metuximab is associated with a significant reduction in the risk of recurrence and death and is well tolerated in patients with unresectable HCC. The combination of TACE and ^131^I-metuximab using the present regimen may postpone relapse in a selected group of patients with unresectable HCC and is an effective palliative treatment option.

## DISCLOSURE

This work was supported by grants from the National Science and Technology Major Project (2012ZX10002-015), the State Key Laboratory of Cancer Biology (CBSKL2019ZZ16), and the Natural Science Foundation of Shaanxi Province (2020SF-252). No other potential conflict of interest relevant to this article was reported.

## Notes

KEY POINTS **QUESTION:** Can ^131^I-metuximab combined with TACE be used as an effective palliative therapy in patients with unresectable HCC? **PERTINENT FINDINGS:** ^131^I-metuximab combined with TACE showed a greater antirecurrence benefit than TACE alone, significantly improved the 5-y survival of patients with advanced HCC over TACE alone, and was well tolerated. **IMPLICATIONS FOR PATIENT CARE:** The combination of TACE and ^131^I-metuximab using the present regimen postpones relapse in a selected group of patients with unresectable HCC and is an effective palliative treatment option.
